# The comparative interrupted time series design for assessment of diagnostic impact: methodological considerations and an example using point-of-care C-reactive protein testing

**DOI:** 10.1186/s41512-022-00118-w

**Published:** 2022-03-02

**Authors:** Thomas R. Fanshawe, Philip J. Turner, Marjorie M. Gillespie, Gail N. Hayward

**Affiliations:** 1 Nuffield Department of Primary Care Health Sciences, University of Oxford, Oxford, OX2 6GG UK; 2 Practice Plus Group, Hawker House, 5-6 Napier Court, Napier Road, Reading, Berkshire, England RG1 8BW UK

**Keywords:** Time series, Quasi-experimental, ARIMA, Diagnostic, C-reactive protein, Point-of-care

## Abstract

**Background:**

In diagnostic evaluation, it is necessary to assess the clinical impact of a new diagnostic as well as its diagnostic accuracy. The comparative interrupted time series design has been proposed as a quasi-experimental approach to evaluating interventions. We show how it can be used in the design of a study to evaluate a point-of-care diagnostic test for C-reactive protein in out-of-hours primary care services, to guide antibiotic prescribing among patients presenting with possible respiratory tract infection. This study consisted of a retrospective phase that used routinely collected monthly antibiotic prescribing data from different study sites, and a prospective phase in which antibiotic prescribing rates were monitored after the C-reactive protein diagnostic was introduced at some of the sites.

**Methods:**

Of 8 study sites, 3 were assigned to receive the diagnostic and 5 were assigned as controls. We obtained retrospective monthly time series of respiratory tract targeted antibiotic prescriptions at each site. Separate ARIMA models at each site were used these to forecast monthly prescription counts that would be expected in the prospective phase, using simulation to obtain a set of 1-year predictions alongside their standard errors. We show how these forecasts can be combined to test for a change in prescription rates after introduction of the diagnostic and estimate power to detect this change.

**Results:**

Fitted time series models at each site were stationary and showed second-order annual seasonality, with a clear December peak in prescriptions, although the timing and extent of the peak varied between sites and between years. Mean one-year predictions of antibiotic prescribing rates based on the retrospective time series analysis differed between sites assigned to receive the diagnostic and those assigned to control. Adjusting for the trend in the retrospective time series at each site removed these differences.

**Conclusions:**

Quasi-experimental designs such as comparative interrupted time series can be used in diagnostic evaluation to estimate effect sizes before conducting a full randomised controlled trial or if a randomised trial is infeasible. In multi-site studies, existing retrospective data should be used to adjust for underlying differences between sites to make outcome data from different sites comparable, when possible.

**Supplementary Information:**

The online version contains supplementary material available at 10.1186/s41512-022-00118-w.

## Background

The development of diagnostic tests is central to improving the timely diagnosis and subsequent treatment of disease. Before a new diagnostic test can become fully established in practice, it is necessary to demonstrate its diagnostic performance in clinical settings, its potential to improve patient outcomes, and its cost-effectiveness [1]. The evaluation cycle from demonstrating analytical performance to cost-effectiveness and broader impact can take a long time: the median time for point-of-care tests has been estimated as 9 years [2], with the range of evidence necessitating a variety of different studies with different designs [3, 4].

Diagnostic accuracy is only a single component in the comprehensive evaluation of a new diagnostic, as recognised in overviews of the field [5, 6], and so it is important to also consider downstream consequences, which might include the effect on treatment prescribing, cost, patient outcomes and adverse effects. These have been collectively termed ‘clinical impact’ [7]. The 2017 European Union regulation on In Vitro Diagnostic Medical Devices (Regulation (EU) 2017/746) specifies that evidence of clinical performance should be demonstrated in order for a CE mark to be gained, a change from the earlier directive 98/79/EC [8].

As such, it has become necessary for studies of new diagnostic devices to include clinical impact measures as outcome variables. Although the randomised controlled trial (RCT) has historically been regarded as the highest quality design for demonstrating the effectiveness of interventions [9], many diagnostic RCTs may be underpowered [10] and the time required can delay the adoption of rapidly evolving technologies, suggesting other designs should be considered.

One such design that has been proposed is the ‘controlled before/after’ study, a quasi-experimental design that can be analysed using methods for comparative interrupted time series (CITS), such as segmented regression. In this design, the diagnostic device can be introduced into a number of locations, and outcomes compared both between the locations using versus those not using the diagnostic; and between the time period after versus the time period before the diagnostic was introduced. As this design partly uses retrospective (so-called ‘real-world’) data, it can reduce the time and cost of conducting such a study, the aim being to provide a plausible estimate of the effect of the diagnostic that can be used subsequently in the design of a full randomised controlled trial of clinical impact.

In medical research, the existing interrupted time series methodology primarily focuses on evaluations of treatments or public health interventions rather than diagnostics [11] and on a single time series from a population rather than multiple time series from different locations [12]. As the CITS design has rarely been used in the evaluation of diagnostics (one example is [13]), there is scope for this design and its associated analytic methods to be explored as a way to evaluate the impact of diagnostics and accelerate the adoption of new technologies into clinical practice.

Point-of-care (POC) diagnostic devices are suitable candidates for evaluations of this form, as they can be introduced to different primary or secondary care services for which relevant clinical impact outcome measures are often already routinely collected. In this paper, we describe the design of study for a POC diagnostic for C-reactive protein (CRP) testing in out-of-hours primary care, and outline how this design affects analytical considerations. Results from the prospective phase of the study will be reported in a subsequent publication.

This paper is structured as follows. First, we give details of the study evaluating POC CRP testing that motivated this work. We then describe a general methodological approach that can be used to design evaluations of this nature, before showing how this was applied to the study in question. The paper concludes with a discussion of relevant issues when using these methods to design studies of diagnostic impact.

## Example: point-of-care C-reactive protein testing

This work was motivated by the design of a study to assess the impact of introducing POC CRP machines to out-of-hours primary care services under the governance of Practice Plus Group. The use of CRP to support antibiotic prescribing decisions for suspected lower respiratory tract infection was supported by the National Institute for Health and Care Excellence Clinical Guideline CG191 (withdrawn after the start of the COVID-19 pandemic) and has been discussed elsewhere [14, 15]. A previous evaluation of out-of-hours primary care services found that as many as 15% of consultations resulted in the issuing of an antibiotic prescription [16], but a systematic review in 2013 estimated a reduction in antibiotic prescribing at consultation in primary care of around 25% when CRP testing had been used [17].

**Fig. 1:**
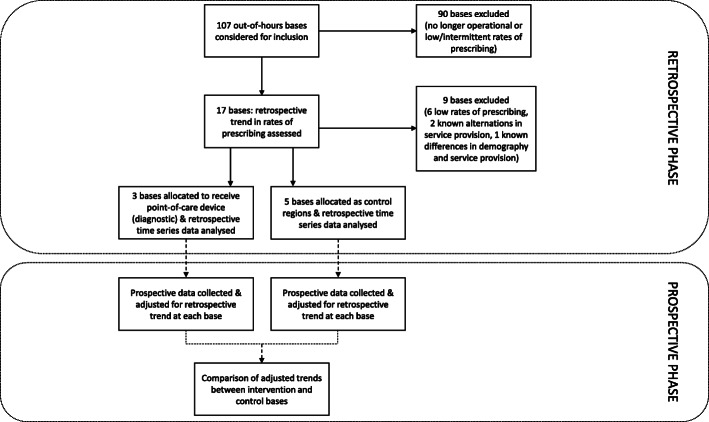
Flow diagram showing the design of the CRP study. Data from the retrospective phase are considered in detail in the current paper

The study aimed to assess the short-term impact of introducing POC CRP machines on antibiotic prescribing in this healthcare setting, with the results potentially being used to inform a longer-term follow-up study or a full cluster randomised controlled trial if there was indication of an improvement in prescribing decisions. It is necessary to obtain an estimate of the effect size for a possible intervention effect as the basis for designing a randomised controlled trial. The CRP study consisted of two phases: a retrospective phase that analysed historic antibiotic prescribing data, and a prospective phase that assessed prescribing data after the introduction of POC CRP machines at certain sites. These machines were provided to OOH clinicians with no restrictions on their clinical use. Guidance on the CRP thresholds above which antibiotics should be considered in patients with suspected lower respiratory tract infection followed NICE guidance at that time. Although many tests were linked to this indication, CRP testing was also used for decision-making in a wider array of clinical contexts, at the discretion of the clinician. The prospective phase used a parallel cluster design, with the periods of measurement at each base coinciding. Figure 1 shows a flow diagram of the whole study design.

Practice Plus Group is contracted to deliver out-of-hours services via a number of primary care ‘bases’ in several regions of England [18]. As the number of machines available for inclusion in the prospective phase of this study was limited to three, an important design decision was how to allocate primary care bases as either receiving POC CRP machines (to perform the diagnostic test), or not receiving machines for POC CRP testing, with the latter group acting as comparators or controls. The choice of sites that received POC CRP machines was made in a non-randomised manner. This decision was informed by examination of the retrospective monthly time series of antibiotic prescription numbers, available separately for each base, and more details are provided in the Results section. The retrospective time series were used in the design to determine the magnitude of change that may have been attributable to the introduction of a POC CRP machine.

The main outcomes were the monthly numbers of respiratory tract targeted antibiotic prescriptions in adults, and total antibiotic prescriptions issued. Therefore, all patients who attended one of the included primary care bases and who was considered for an antibiotic prescription could potentially contribute data. A list of included respiratory tract targeted antibiotics appears in the Additional file 1. Secondary outcomes (not discussed further in the current paper) included total non-topical antibiotic prescriptions, the proportion of patients requiring further general practitioner contact or hospital admission within 14 days, the time required for testing and the test failure rate. A qualitative substudy, also to be reported elsewhere, aimed to explore clinicians’ perspectives of the use of POC CRP tests in out-of-hours services.

## Methods: adaptation of comparative interrupted time series design

The CITS design is an extension of the interrupted time series design that has been widely used as a quasi-experimental approach for the evaluation of health policies or other interventions for which randomisation may be infeasible, such as those in education settings [19–22].

Some papers have investigated sample size and power considerations for these types of designs. Cruz et al. described power considerations for interrupted time series models, but their model was aimed at change-point detection, which is less relevant when the time of introducing a diagnostic test is known [23]. Zhang et al. examined the relationship between power and the number of time-points in the available time series, also for a single time series, and they restricted their model to be of autoregressive (AR and ARCH) form [24].

The general ARMA(p,q), or autoregressive moving average, model for the time series (*y*_*t*_ : *t* = 1, …, *n*) has the form

1 $$ {\displaystyle \begin{array}{c}{y}_t=\delta +\left({\phi}_1{y}_{t-1}+\dots +{\phi}_p{y}_{t-p}\right)+\left({\theta}_1{\epsilon}_{t-1}+\dots +{\theta}_q{\epsilon}_{t-q}\right)+{\epsilon}_t\\ {}\ \end{array}} $$

In this equation, *δ* represents the mean level of the outcome (y), (*ϕ*_1_, …, *ϕ*_*p*_) are parameters that reflect its dependence on previous values of the time series (the autoregressive component), (*ϵ*_1_, …*ϵ*_*n*_) are random variables that are assumed to be independent errors, and (*θ*_1_, …, *θ*_*q*_) are parameters that reflect the dependence of the time series on previous error terms (the moving average component). The parameter *δ* is assumed constant in the formulation above but can be supplemented with another functional form, such as a linear or non-linear time trend, if required.

In this paper, we use the more general ARIMA(p,d,q), or autoregressive integrated moving average model, which extends [1] to allow for situations in which the time series is not stationary (i.e. if the assumption that its mean, variance and autocorrelation do not fluctuate over time does not hold). Further details of the models are provided in the Additional file 1 and Chapters 3.4.6 and 4.6 of the book by Chatfield [25].

Alternatives to these models include simpler linear models that may not allow for autocorrelation [19, 26], and dynamic models that model this autocorrelation via correlated, temporally evolving random processes [27].

A flexible implementation of this class of models is provided by the automatic ARIMA time series package for R, which selects a best-fitting model among the class required using the Akaike Information Criterion or Bayesian Information Criterion [28].

Methods of prediction from ARIMA models for forecasting individual values of *y*_*t*_ for *t* ≥ *n* + 1 using the Kalman filter have previously been described [29, 30] and implemented in the simulate.ets() function in the R ‘forecast’ package [28, 31]. For the purpose of the present work, interest lies in simulating values of ${S}_k={\sum}_{t=n+1}^{n+k}{\hat{y}}_t$, where ${\hat{y}}_t$ are forecasted values of the time series and, for example, *k* = 12 if *t* represents time in months and the follow-up period is scheduled to last for 1 year. Thus *S*_*k*_ represents the sum of forecasted values over the subsequent year. In such a case, the ${\hat{y}}_t$ will typically be positively correlated, and using the mean and standard error of the predictive distributions of each ${\hat{y}}_t$ independently to estimate the standard error of *S*_*k*_ will underestimate the latter if this correlation is not accounted for. Instead, the mean and standard error of the predictive distribution of *S*_*k*_ can be estimated by repeated direct simulation: simulating a complete vector $\left({\hat{y}}_t:t=n+1,\dots, n+k\right)$, using the sum as a single estimate of *S*_*k*_, repeating, and then calculating the mean ${\hat{m}}_k$ and standard deviation ${\hat{s}}_k$ over all calculated estimates of *S*_*k*_.

After observing the follow-up data values (*y*_*t*_ : *t* = *n* + 1, …, *n* + *k*), a standardised measure of the increase in observed values over the expected values based on the retrospective time series can be calculated as

2 $$ Z=\frac{\sum_{t=n+1}^{n+k}{y}_t-{\hat{m}}_k}{{\hat{s}}_k} $$

Tests can be combined as a global *z*-test using standard methods [32], treating the individual test statistics as realisations from a Normal distribution with known mean 0 and variance 1. In a study with *n* intervention regions and m control regions, if ${\overline{Z}}_I$ and ${\overline{Z}}_C$ are the means of the *Z*-statistics in the intervention regions and the control regions, respectively, then a test statistic for the difference in means is

3 $$ \frac{{\overline{Z}}_I-{\overline{Z}}_C}{\sqrt{n^{-1}+{m}^{-1}}} $$

Equations (2) and (3) allow estimation of the power to detect a change in the number of prescriptions relative to the trend in the retrospective time series. Consider a test for a single site, as given by [2].If $V={\sum}_{t=n+1}^{n+k}{y}_t$ follows a Normal distribution with mean *m*^∗^ and standard deviation *s*^∗^, then a hypothesis test of size *α* based on [2] will detect a reduction from the trend based on the retrospective time series if $V<{\hat{m}}_k-{\hat{s}}_k\ {\Phi}^{-1}\left(1-\alpha /2\right)$, where Φ(*z*) is the cumulative distribution function of the standard Normal distribution. This occurs with probability (power)

4 $$ \Phi \left(\frac{\left({\hat{m}}_k-{m}^{\ast}\right)-{\hat{\mathrm{s}}}_k\ {\Phi}^{-1}\left(1-\alpha /2\right)}{s^{\ast }}\right) $$

Derivation of (3) and (4) is shown in the Additional file 1.

## Results

To adapt the method described above to the POC CRP study, we first obtained the time series of respiratory tract targeted antibiotic prescription data for each of the 107 out-of-hours bases that were candidates for inclusion, and examined these graphically. The majority of these (90 bases) could be immediately excluded from consideration either because service alterations meant that the base was no longer in operation, or because prescription counts were extremely low or variable over time, and therefore unlikely to be comparable with those from larger bases.

**Fig. 2:**
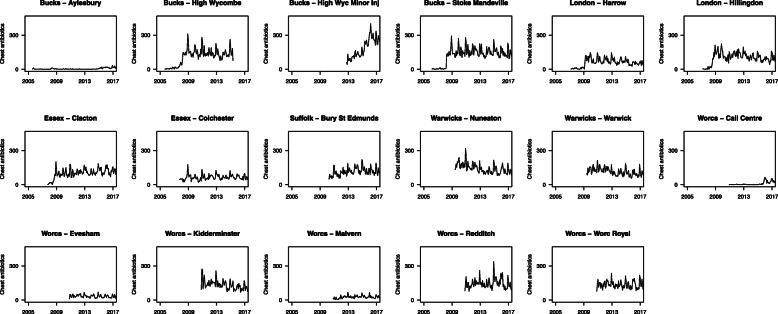
Retrospective time trends for respiratory tract targeted antibiotic prescribing for 17 candidate bases for inclusion

The time trends for the remaining 17 bases (Fig. 2) were further assessed by two of the authors (TF and PT), and nine of these were subsequently excluded, either because counts were judged too low to be comparable with the remainder (Aylesbury, Harrow, Colchester, Worcester Call Centre, Evesham, Malvern), because of known alterations to service provision that resulted in a markedly unusual time trend (the two High Wycombe bases), or because the demography of users and service provision in London (Hillingdon) was thought to be non-comparable with that in sites outside London. Of the remaining eight bases, three (Kidderminster, Redditch, Worcestershire Royal) were allocated to receive the point-of-care device, and five were assigned as control bases (Stoke Mandeville, Clacton, Bury St. Edmunds, Nuneaton, Warwick). This was a pragmatic, non-randomised allocation as it was desirable that all bases receiving point-of-care devices should lie within the same administrative region (Worcestershire).

**Fig. 3:**
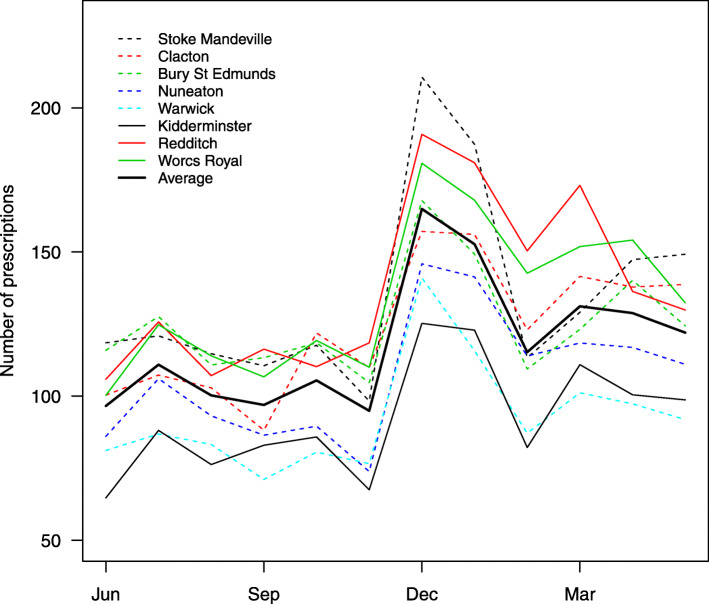
Average monthly numbers of respiratory tract targeted antibiotic prescriptions for the eight included bases and their overall average, during the retrospective phase of the study. Bases receiving a POC CRP machine are shown as solid lines, and those not receiving a machine as dashed lines

There was no clearly consistent increasing or decreasing trend across all eight retained bases, but rather some variation between bases in the nature of the trend and the level of total prescribing, suggesting that separate models for each time series may be appropriate. There was seasonality in prescribing rates, with a clear winter peak and in particular a spike in prescribing rates during December (Fig. 3).

Table 1 shows the form of the fitted resulting time series models at each base. Autocorrelation function plots used to check the model fit are available in the Additional file 1. The fitted models show no evidence of non-stationarity in the time series at any of the bases. Each shows second-order annual seasonality, suggesting correlation of the seasonal peak with those in the two previous years, and at most first order autocorrelation or correlated errors in the deseasonalised time series. Autocorrelation functions plots for the eight original time series, and for the residuals from the fitted models, show that the models adequately account for the autocorrelation in the original time series (Additional file 1, Supplementary Fig. 1).

**Table 1:** Estimated parameter values from fitting separate ARIMA(*p,d,q*)(*P,D,Q*)[*M*] to the retrospective time series at each site

Base	*p*	*d*	*q*	*P*	*D*	*Q*	Period, *M*
Stoke Mandeville	0	0	0	2	0	0	12
Clacton	0	0	0	2	0	0	12
Bury St Edmunds	1	0	0	2	0	0	12
Nuneaton	0	0	1	2	0	0	12
Warwick	1	0	1	2	0	0	12
Kidderminster	0	0	1	2	0	0	12
Redditch	1	0	1	2	0	0	12
Worcestershire Royal	0	0	0	2	0	0	12

Here, *p* is the order of the autoregressive term, *q* is the order of the moving average term, and *d* the degree of differencing, with *P*, *Q*, and *D* defined similarly in relation to a seasonal component of period *M* months (see Additional file 1 for details).

**Fig. 4:**
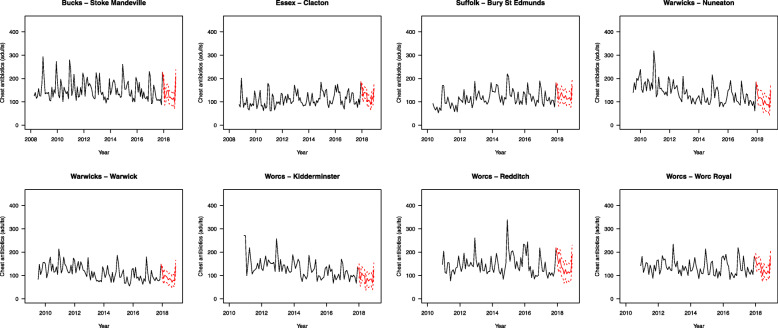
Retrospective time trends for respiratory tract targeted antibiotic prescribing (black lines) with 12-month predicted values (solid red lines) ± one standard error (dashed red lines), for the eight included bases

Figure 4 shows forecasts for an additional 12 months for all eight bases, and shows variation between the bases in their forecasted monthly values.

Of note, the mean annual expected number of prescriptions in the three intervention bases was 1452, while the mean number in the five control bases was only 1401, whereas if the intervention has no impact on prescribing rates compared to control, by (2) the expected change in the prescribing rates between the retrospective and the prospective phases at each site is 0. Under the same assumption, the expected value of (4) is also 0.

**Fig. 5:**
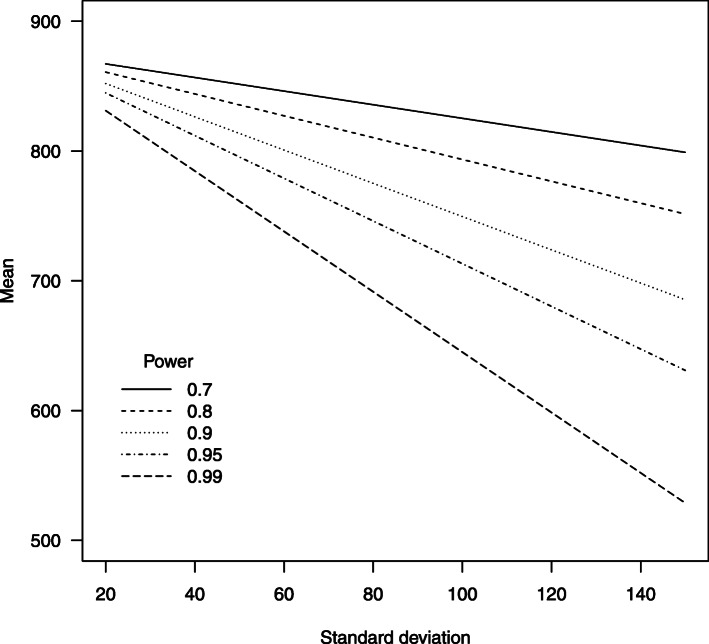
Contours of constant power (at *α* = 0.05) to detect a decrease from the trend in the retrospective time series at the Kidderminster site, for different values of the mean and standard deviation of the number of prescriptions during the prospective study phase

As an example to illustrate the power function (4), consider a test for the Kidderminster site based on the simulated mean and standard deviation values shown in Table 2. Figure 5 shows pairs (*m*^∗^, *s*^∗^) that are consistent with equivalent values of the power [4] (if *α* = 0.05). For example, if *s*^∗^ = 100, the mean of the distribution of prescriptions occurring at Kidderminster during the 12-month follow-up period would need to be around 740 (a decrement of around 367 prescriptions compared with the predicted mean from Table 2) to reach 90% power. Under the same conditions, for Redditch a larger reduction would be required (to 1070 prescriptions, a decrement of 572), as a result of the larger monthly variability at that site, as reflected by the value of ${\hat{s}}_{12}$.

**Table 2:** Estimated mean and standard deviation of 12-month forecasted number of prescriptions calculated without (columns 2 and 3) and with (columns 4 and 5) allowance for correlation in forecasted values

	Directly calculated		Simulated (allowing for correlation)	
Base	${\hat{m}}_{12}$	${\hat{s}}_{12}$	${\hat{m}}_{12}$	${\hat{s}}_{12}$
Stoke Mandeville	1618	97	1615	98
Clacton	1485	82	1484	82
Bury St Edmunds	1505	99	1505	116
Nuneaton	1283	111	1281	135
Warwick	1114	84	1112	130
Kidderminster	1106	99	1107	117
Redditch	1645	133	1642	226
Worcestershire Royal	1605	93	1606	92

## Discussion

This paper has outlined a method for designing studies for evaluating clinical impact of diagnostics by using a combination of retrospective and prospectively collected data as part of a CITS design. This approach has the advantage of enabling an estimate of a plausible effect size to be obtained, at relatively low resource, with the view of carrying out a larger randomised study if it is feasible to do so.

The design strategy outlined here uses retrospective data on prescribing rates as a means of directly adjusting for between-site differences. It is recommended to obtain the retrospective data before initiating the prospective phase, as the current paper has shown that it can be used both to inform an appropriate allocation of sites as intervention or comparator regions, and to estimate statistical power. Having access to relevant retrospective data at the planning stage should therefore be seen as highly beneficial, although not mandatory, if choosing to adopt a CITS design of this type.

Our approach adjusts for the retrospective trend at each site in order to make sites more comparable when analysing their data from the prospective phase. Adjusting for the modelled monthly time trend enables the effect of introducing the diagnostic to be estimated without bias, as in the absence of any effect the expected value of this estimate is zero. An alternative approach would be to attempt to collect site-specific covariates, or covariates that reflect differences in characteristics of patients attending the different sites, to perform an adjustment between sites. In the present study it is unlikely that a sufficient set of covariates could be found to eliminate these differences, and little patient-specific information was available in the routinely-collected dataset. The trend seen in the retrospective phase can therefore be seen as a proxy for a variety of unknown characteristics that are particular to the site, the patients attending the site, and the clinicians making prescribing decisions, all of which might affect the observed prescribing rates. A more detailed discussion of the choice of comparator regions in designs of this type is available elsewhere [22].

The strong seasonality shown in antibiotic prescribing rates has been previously noted in studies in Europe [33] and in out-of-hours services and general practice more broadly in the UK [34]. While to a large extent this reflects the seasonal nature of presentation of patients with symptoms of respiratory tract infection [35], the clear December peak observed here may reflect a shift from booked general practice appointments to out-of-hours appointments resulting from seasonal general practice service restriction or closure. As our application concerned antibiotic prescribing, allowing for seasonality was important, but the method is generalisable to any outcome that might be measured via a CITS design, provided a suitable model for the time series in question is used.

This investigation has some limitations. As previously noted, the approach using observational data outlined here is not intended as a replacement for a well-conducted and adequately-powered RCT, in situations in which performing an RCT is feasible. In the absence of randomisation, systematic between-site differences or ‘rising tide’ effects that might influence prescribing rates cannot be ruled out. However, as site-specific retrospective trends are adjusted for in the analytic approach, the effect of these confounding differences would need to change over time, differentially between sites with and without the diagnostic, to affect conclusions. In this respect, the CITS design appears stronger than both a non-randomised before-after design that lacks control regions, and a non-randomised design that uses data from the prospective data collection period alone. Recent research suggests that in many scenarios, well-conducted CITS studies may give results that are comparable to those from intervention RCTs [26, 36], and it should be noted further that ‘test-treatment’ randomised trials of diagnostics may themselves be subject to bias or other methodological limitations [37].

In this paper we have concentrated on the analysis of the primary outcome, whereas in practice a variety of other outcomes would typically need to be considered, including costs associated with adopting the diagnostic. Overall antibiotic prescribing rates, which have been shown to be high in OOH care [38], can be readily monitored and compared between different sites, but do not reflect the success or otherwise of antibiotic treatment for individual patients. We have also considered a single class of models for the retrospective time series. Upon completion of the study, the analysis of the prospective data component may be more nuanced than that described here: models for CITS data may allow for both a step change and a change in trend or gradient, among others [19], and a suitable functional form is difficult to specify in advance, especially since this may vary between sites. We intend to explore these issues when reporting the results of the prospective phase of the study.

Previously, quasi-experimental studies have been used more often for assessing interventions than for diagnostic impact. They have gained particular popularity for studies of policy changes that are not amenable for performing RCTs. The deployment of non-randomised studies aligns with the ongoing Impact Health Technology Assessment project (www.impact-hta.eu/work-package-6), which aims to compare treatment effects of interventions between randomised and non-randomised studies, and the Innovation Medicine Initiative GetReal project (www.imi-getreal.eu), which uses real-world information for drug development. Comparably, the Cancer Drug Fund provides resources for faster introduction of new cancer treatments alongside evaluation of their clinical and cost-effectiveness by collecting data for the evaluation of new drugs during the implementation period.

More rapid assessment of novel diagnostics remains a research priority, and the methods described in this paper outline one possible approach. Of further interest would be an adaptation to simultaneously monitor diagnostic accuracy measures, such as sensitivity and specificity, while also evaluating clinical impact, as these steps are often currently performed as part of separate studies. This may be suitable for diagnostics that have already met requirements for regulatory approval but require ongoing assessment of diagnostic accuracy performance as an element of regulatory post-market surveillance, for example, or those that are being considered for use in different populations. A comparison with other quasi-experimental designs, such as stepped wedge designs for RCTs, in which the time when the diagnostic is introduced differs in different locations, may also be of value.

## Conclusions

The method outlined here can be used in quasi-experimental designs for diagnostic evaluation. In such studies, models should adjust for underlying trends in outcomes, especially in multi-site studies, for which existing retrospective data can be used. This approach can be beneficial in the evaluation of diagnostic impact, which provides essential evidence in the pathway for bringing new diagnostic devices into clinical practice.

## Supplementary Information

**Additional file 1.** List of respiratory tract targeted antibiotics and figure of autocorrelation plots.

## Data Availability

The data that support the findings of this study were provided by Practice Plus Group but restrictions apply to the availability of these data, which were used under license for the current study, and so are not publicly available. Data are however available from the authors upon reasonable request and with permission of relevant stakeholders.

## Abbreviations

AR: Auto-regressive
ARCH: Auto-regressive conditional heteroscedasticity
ARIMA: Auto-regressive integrated moving average
ARMA: Auto-regressive moving average
CITS: Comparative interrupted time series
CRP: C-reactive protein
POC: Point-of-care
RCT: Randomised controlled trial

## References

[1] Lord SJ, Irwig L, Simes RJ (2006). When is measuring sensitivity and specificity sufficient to evaluate a diagnostic test, and when do we need randomized trials. Ann Intern Med..

[2] Verbakel JY, Turner PJ, Thompson MJ, Plüddemann A, Price CP, Shinkins B, van den Bruel A (2017). Common evidence gaps in point-of-care diagnostic test evaluation: a review of horizon scan reports. BMJ Open..

[3] Shinkins B, Yang Y, Abel L, Fanshawe TR (2017). Evidence synthesis to inform model-based cost-effectiveness evaluations of diagnostic tests: a methodological review of health technology assessments. BMC Med Res Methodol..

[4] Yang Y, Abel L, Buchanan J, Fanshawe T, Shinkins B (2019). Use of decision modelling in economic evaluations of diagnostic tests: an appraisal and review of Health Technology Assessments in the UK. Pharmacoecon Open..

[5] Bossuyt PM, Lijmer JG, Mol BW (2000). Randomised comparisons of medical tests: sometimes invalid, not always efficient. Lancet..

[6] Van den Bruel A, Cleemput I, Aertgeerts B, Ramaekers D, Buntinx F (2007). The evaluation of diagnostic tests: evidence on technical and diagnostic accuracy, impact on patient outcome and cost-effectiveness is needed. J Clin Epidemiol..

[7] Steyerberg EW, Moons KG, van der Windt DA, Hayden JA, Perel P, Schroter S (2013). Prognosis Research Strategy (PROGRESS) 3: prognostic model research. PLoS Med..

[8] Regulation (EU) 2017/746 of the European Parliament and of the Council, L117/176. https://eur-lex.europa.eu/legal-content/EN/TXT/HTML/?uri=CELEX:32017R0746&from=EN. Accessed 11 June 2020.

[9] Deeks JJ, Dinnes J, D'Amico R, Sowden AJ, Sakarovitch C, Song F (2003). Evaluating non-randomised intervention studies. Health Technol Assess.

[10] Ferrante di Ruffano L, Deeks JJ (2016). Test-treatment RCTs are sheep in wolves' clothing. J Clin Epidemiol.

[11] Wagner AK, Soumerai SB, Zhang F, Ross-Degnan D (2002). Segmented regression analysis of interrupted time series studies in medication use research. J Clin Pharm Ther..

[12] Bernal JL, Cummins S, Gasparrini A (2017). Interrupted time series regression for the evaluation of public health interventions: a tutorial. Int J Epidemiol..

[13] Bjerrum L, Cots JM, Llor C, Molist N, Munck A (2006). Effect of intervention promoting a reduction in antibiotic prescribing by improvement of diagnostic procedures: a prospective, before and after study in general practice. Eur J Clin Pharmacol..

[14] Cals JW, Ebell MH (2018). C-reactive protein: guiding antibiotic prescribing decisions at the point of care. Br J Gen Pract..

[15] Ward C (2018). Point-of-care C-reactive protein testing to optimise antibiotic use in a primary care urgent care centre setting. BMJ Open Quality..

[16] Hayward GN, Fisher RFR, Spence GT, Lasserson DS (2016). Increase in antibiotic prescriptions in out-of-hours primary care in contrast to in-hours primary care prescriptions: service evaluation in a population of 600 000 patients. J Antimicrob Chemoth..

[17] Huang Y, Chen R, Wu T, Wei X, Guo A (2013). Association between point-of-care CRP testing and antibiotic prescribing in respiratory tract infections: a systematic review and meta-analysis of primary care studies. Br J Gen Pract..

[18] Out-of-hours services (OOH). https://www.careukhealthcare.com/our-services/out-of-hours-services. Accessed 11 June 2020.

[19] Kontopantelis E, Doran T, Springate DA, Buchan I, Reeves D (2015). Regression based quasi-experimental approach when randomisation is not an option: interrupted time series analysis. BMJ..

[20] Hudson J, Fielding S, Ramsay CR (2019). Methodology and reporting characteristics of studies using interrupted time series design in healthcare. BMC Med Res Methodol..

[21] Biglan A, Ary D, Wagenaar AC (2000). The value of interrupted time-series experiments for community intervention research. Prev Sci..

[22] Jacob R, Somers M-A, Zhu P, Bloom H (2016). The validity of the comparative interrupted time series design for evaluating the effect of school-level interventions. Evaluation Rev..

[23] Cruz M, Gillen DL, Bender M, Ombao H (2019). Assessing health care interventions via an interrupted time series model: Study power and design considerations. Stat Med..

[24] Zhang F, Wagner AK, Ross-Degnan D (2011). Simulation-based power calculation for designing interrupted time series analyses of health policy interventions. J Clin Epidemiol..

[25] Chatfield C (2003). The analysis of time series: an introduction.

[26] Fretheim A, Zhang F, Ross-Degnan D, Oxman AD, Cheyne H, Foy R, Goodacre S, Herrin J, Kerse N, McKinlay RJ, Wright A, Soumerai SB (2015). A reanalysis of cluster randomized trials showed interrupted time-series studies were valuable in health system evaluation. J Clin Epidemiol..

[27] Fanshawe TR, Diggle PJ, Rushton S, Sanderson R, Lurz PWW, Glinianaia SV, Pearce MS, Parker L, Charlton M, Pless-Mulloli T (2008). Modelling spatio-temporal variation in exposure to particulate matter: a two-stage approach. Environmetrics..

[28] Hyndman RJ, Khandakar Y (2008). Automatic time series forecasting: the forecast package for R. J Stat Softw..

[29] Harvey AC, McKenzie CR (1982). Algorithm AS 182: finite sample prediction from ARIMA processes. J R Stat Soc C - App..

[30] Durbin J, Koopman SJ (2012). Time series analysis by state space methods.

[31] Hyndman R, Athanasopoulos G, Bergmeir C, Caceres G, Chhay L, O'Hara-Wild M, et al. forecast: Forecasting functions for time series and linear models. R package version 8.12. 2020. https://pkg.robjhyndman.com/forecast.

[32] Weatherburn CE (1968). A first course in mathematical statistics.

[33] Elseviers MM, Ferech M, Vander Stichele RH, Goossens H, ESAC Project Group (2007). Antibiotic use in ambulatory care in Europe (ESAC data 1997–2002): trends, regional differences and seasonal fluctuations. Pharmacoepidem Dr S..

[34] Curtis HJ, Walker AJ, Mahtani KR, Goldacre B (2018). Time trends and geographical variation in prescribing of antibiotics in England 1998–2017. J Antimicrob Chemoth..

[35] Fleming DM, Ross AM, Cross KW, Kendall H (2003). The reducing incidence of respiratory tract infection and its relation to antibiotic prescribing. Br J Gen Pract..

[36] St. Clair T, Cook TD, Hallberg K (2014). Examining the internal validity and statistical precision of the comparative interrupted time series design by comparison with a randomized experiment. Am J Eval..

[37] Ferrante di Ruffano L, Dinnes J, Sitch AJ, Hyde C, Deeks JJ (2017). Test-treatment RCTs are susceptible to bias: a review of the methodological quality of randomized trials that evaluate diagnostic tests. BMC Med Res Methodol.

[38] Edelstein M, Agbebiyi A, Ashiru-Oredope D, Hopkins S (2017). Trends and patterns in antibiotic prescribing among out-of-hours primary care providers in England, 2010–14. J Antimicrob Chemoth..

