# Supplementary material for: The comparative interrupted time series design for assessment of diagnostic impact: methodological considerations and an example using point-of-care C-reactive protein testing
Source: Diagn Progn Res. 2022 Mar 2;6:3. doi: 10.1186/s41512-022-00118-w (PMC8888027; doi:10.1186/s41512-022-00118-w)
Supplement: Supplementary file 1 — Additional file 1. List of respiratory tract targeted antibiotics and figure of autocorrelation plots. [file 41512_2022_118_MOESM1_ESM.docx]

**Supplementary Material**

List of respiratory tract targeted antibiotics (initially obtained using the British National Formulary in conjunction with prescription lists from the observed data files, and reviewed by practising general practitioners who had access to local and national antimicrobial prescribing guidelines):

Amoxicillin

Amoxil

Augmentin

Augmentin-duo

Azithromycin

Cefalexin

Clarithromycin

Co-amoxiclav

Doxycycline

Erythrocin

Erythrolar

Erythromycin

Erythroped

Oxytetracycline

Tetracycline

**Further details of ARIMA model**

The general ARIMA(p,d,q) model extends model [1] and arises from taking d degrees of differencing in order to make the resulting differenced time series stationary. For prescribing data we anticipate seasonality, with more prescriptions being made during the winter, and therefore extend this ARIMA model to allow period M (months) seasonality, written ARIMA(p,d,q)(P,D,Q)[M], i.e. also allowing the seasonal trend to follow an ARIMA model, and anticipating M=12. A model with $d=D=0$ reflects a stationary time series, in which case the model reverts to ARMA form.

**Derivation of equations [3] and [4]**

By distributional assumption, the test statistic from a single site ($Z$ in equation [2]) follows a Notmal distribution with mean 0 and variance 1. Given n intervention regions and m control regions, assumed to be independent of each other, if $\bar{Z}_{I}$ and $\bar{Z}_{C}$ are the means of the Z-statistics in the intervention regions and the control regions, respectively, then by usual properties of the Normal distribution, $\bar{Z}_{I} \sim N(0,n^{-1})$ and $\bar{Z}_{C} \sim N(0,m^{-1})$. Thus $\bar{Z}_{I}-\bar{Z}_{C} \sim N(0,{n^{-1}+m}^{-1})$ and

$$\frac{\bar{Z}_{I}-\bar{Z}_{C}}{\sqrt{n^{-1}+m^{-1}}}\sim N(0,1)$$

and can therefore be used as a test statistic based on the standard Normal distribution.

For a single site, using the test statistic $Z$ in equation [2], a hypothesis test of size $\alpha$ will detect a reduction from the assumed underlying trend if $Z<\Phi^{-1}(1-\alpha/2)$, i.e. if $V<\hat{m}_{k}-{\hat{s}_{k} \Phi}^{-1}(1-\alpha/2)$, where $V=\sum_{t=n+1}^{n+k} y_{t}$. Given that $V \sim N(m^{*},s^{*2}$), the probability that this reduction will be detected, and therefore the power, is given by equation [4].

**Supplementary Figure 1**

Autocorrelation function plots for each of the eight included bases, for the original data (top line) and for the residuals from the fitted time series model (bottom line). Columns, from left to right, show data from Stoke Mandeville; Clacton; Bury St Edmunds; Nuneaton; Warwick; Kidderminster; Redditch; Worcestershire Royal.
